# Comparative one-month safety and effectiveness of five leading new-generation devices for transcatheter aortic valve implantation

**DOI:** 10.1038/s41598-019-53081-w

**Published:** 2019-11-19

**Authors:** Arturo Giordano, Nicola Corcione, Paolo Ferraro, Alberto Morello, Sirio Conte, Luca Testa, Francesco Bedogni, Alessandro Iadanza, Sergio Berti, Damiano Regazzoli, Enrico Romagnoli, Carlo Trani, Francesco Burzotta, Martino Pepe, Giacomo Frati, Giuseppe Biondi-Zoccai, The Registro Italiano GISE sull’impianto di Valvola Aortica Percutanea (RISPEVA) Study Investigators, Corrado Tamburino, Federica Ettori, Anna Sonia Petronio, Mauro Rinaldi, Silvio Klugmann, Paolo Rubino, Elena Tremoli, Alfredo Marchese, Gennaro Sardella, Gianfranco Pistis, Elvis Brscic, Pietro Giudice, Luigi Piatti, Diego Ardissino, Ciro Indolfi, Francesco Chiarella, Maurizio Tespili, Stefano De Servi, Roberto Bonmassari, Antonio Fappani, Claudio Cuccia, Alberto Cremonesi, Fabrizio Tomai

**Affiliations:** 1 Unita‘ Operativa di Interventistica Cardiovascolare, Pineta Grande Hospital, Castel Volturno, Italy; 2 Unità Operativa di Emodinamica, Santa Lucia Hospital, San Giuseppe Vesuviano, Italy; 3 Department of Cardiology, IRCCS Policlinico San Donato, San Donato Milanese, Milan, Italy; 4 Divisione di Emodinamica, Dipartimento di Scienze Cardiache, Toraciche e Vascolari, Policlinico Santa Maria alle Scotte, Siena, Italy; 5 Fondazione C.N.R. G. Monasterio Ospedale del Cuore, Massa, Italy; 6 Department of Biomedical Sciences, Humanitas University, Rozzano, Italy; 7 Institute of Cardiology, Fondazione Policlinico Universitario A. Gemelli IRCCS, Università Cattolica del Sacro Cuore, Rome, Italy; 8 Division of Cardiology, Department of Emergency and Organ Transplantation, University of Bari, Bari, Italy; 9 Department of Medico-Surgical Sciences and Biotechnologies, Sapienza University of Rome, Latina, Italy; 10 IRCCS NEUROMED, Pozzili, Italy; 11 Mediterranea Cardiocentro, Napoli, Italy; 12 University of Catania, Catania, Italy; 13 Spedali Civili di Brescia, Brescia, Italy; 14 University of Pisa, Pisa, Italy; 15 University of Turin, Turin, Italy; 16 Ospedale Niguarda Ca’ Granda, Milan, Italy; 17 Clinica Montevergine, Avellino, Italy; 18 Centro Cardiologico Monzino, Milan, Italy; 19 Anthea Hospital GVM Care&Research, Bari, Italy; 20 Sapienza University of Rome-Policlinico Umberto I, Rome, Italy; 21 “SS Antonio e Biagio e Cesare Arrigo” Hospital, Alessandria, Italy; 22 Maria Pia Hospital, Turin, Italy; 23 San Giovanni di Dio e Ruggi d’Aragona, Salerno, Italy; 24 Ospedale di Lecco, Lecco, Italy; 25 Azienda Ospedaliera Universitaria di Parma, Parma, Italy; 26 Policlinico Universitario Mater Domini, Catanzaro, Italy; 27 IRCCS San Martino, Genova, Italy; 28 Ospedale Bolognini, Seriate, Italy; 29 Ospedale Civile di Legnano, Legnano, Italy; 30 Ospedale S. Chiara, Trento, Italy; 31 Istituto Clinico San Rocco, Ome, Italy; 32 Fondazione Poliambulanza, Brescia, Italy; 33 Maria Cecilia Hospital, Cotignola, Italy; 34 European Hospital, Rome, Italy

**Keywords:** Interventional cardiology, Medical research

## Abstract

Transcatheter aortic valve implantation (TAVI) for aortic stenosis is becoming an appealing alternative to surgical aortic valve replacement in high-risk patients and to medical therapy for inoperable ones. Several new-generation TAVI devices have been recently introduced, but comparative analyses are lacking. We aimed to compare 1-month outcomes associated with such five leading new-generation TAVI devices exploiting data collected in the prospective observational RISPEVA (Registro Italiano GISE sull’impianto di Valvola Aortica Percutanea) Study. We queried the dataset of the ongoing RISPEVA study to retrieve baseline, procedural and 1-month outcome details of patients undergoing TAVI with Acurate, Evolut, Portico, Lotus, and Sapien3. Analysis was based on unadjusted and propensity score-adjusted methods. We included 1976 patients, 234 (11.8%) treated with Acurate, 703 (35.6%) with Evolut, 151 (7.6%) with Lotus, 347 (17.6%) with Portico, and 541 (27.4%) with Sapien3. Unadjusted analysis for baseline features highlighted several significant differences, and other discrepancies were found for procedural features. Despite these differences, device and procedural success were similarly high (ranging from 98.0% to 99.4%, p > 0.05). However, procedural valve migration appeared more common with Acurate (p = 0.007), and major bleeding with Sapien3 (p = 0.002). Unadjusted analysis for 1-month outcomes also highlighted significant differences in the composite of death, stroke, myocardial infarction, major vascular complication, major bleeding, or renal failure (favoring Portico, p < 0.001), major vascular complications (favoring Lotus, p < 0.001), renal failure (favoring Portico, p = 0.035), and permanent pacemaker implantation (favoring Acurate, p < 0.001). Propensity score-adjusted analyses showed lower rates of major adverse events with Evolut and Portico (p < 0.05), major vascular complications with Lotus and Portico (p < 0.05), renal failure with Sapien3 (p < 0.05) and permanent pacemaker implantation with Acurate (p < 0.05). In conclusion, new-generation TAVI devices have different profiles of early comparative safety and efficacy. These findings should be taken into account for individualized decision making and patient management.

## Introduction

The management of aortic stenosis has been revolutionized by the introduction of transcatheter aortic valve implantation (TAVI) less than 20 years ago^1^. After the first pioneering efforts, several landmark trials using first-generation devices established the superiority of TAVI in comparison to medical therapy encompassing valvuloplasty in patients at prohibitive surgical risk and the non-inferiority of TAVI in comparison to surgical aortic valve replacement in subjects at high or intermediate risk, and, most recently, in low-risk patients^2,3^. Despite these breakthroughs, uncertainty persists on the long-term durability of TAVI and the precise impact of residual aortic regurgitation. Indeed, very long-term (>10 years) follow-up will be required to accurately gauge these risks^1^. Awaiting such strategic data, another area of controversy is the comparison between different TAVI devices.

While all new-generation devices boast important technological refiments including reduced size and skirt to minimize paravalvular leak, there are now at least 7 different TAVI devices available for clinical use^1^. Whereas Sapien3 is the only balloon-expandable valve, even among self-expandable devices, which include Acurate, Allegra, Evolut, JenaValve, Lotus, and Portico, differences abound^4,5^. For instance, manufacturers have diversified their products with alternative choices for device length, strut thickness, cell size, radial force, skirt length, and delivery method. To date, few comparative studies have been reported on TAVI devices. In particular, Vlaastra and colleagues showed that Sapien3 may be associated with lower surgical conversion rates, fewer strokes and permanent pacemaker implantations (PPI), but more bleedings than Evolut^6^, whereas Pagnesi *et al*. have reported similar short-term results with Acurate and Evolut^4^. Other comparative analyses have provided hints at other possible differences as well^7–9^. Of course, ongoing randomized trials will provide more accurate outcome data, but in the meanwhile, we aimed to inform clinicians and patients on the short-term comparative effectiveness and safety of 5 leading TAVI devices using the clinical data prospectively collected in the Registro Italiano GISE sull’impianto di Valvola Aortica Percutanea (RISPEVA) study^10^.

## Methods

### Design

The RISPEVA Study has been previously described in detail elsewhere^10–12^. Briefly, RISPEVA was a national prospective observational study focusing on TAVI conducted in several Italian centers. All methods were carried out in accordance with relevant guidelines and regulations. All experimental protocols were approved by the institutional ethics committee of: Policlinico San Donato, San Donato Milanese, Milan; Pineta Grande Hospital, Castel Volturno; Policlinico Santa Maria alle Scotte, Siena; Fondazione C.N.R. G. Monasterio Ospedale del Cuore, Massa; Humanitas University, Rozzano;, Fondazione Policlinico Universitario A. Gemelli IRCCS, Rome; University of Catania, Catania; Spedali Civili di Brescia, Brescia; University of Pisa, Pisa; University of Turin, Turin; Ospedale Niguarda Ca’ Granda, Milan; Clinica Montevergine, Avellino; Centro Cardiologico Monzino, Milan; Anthea Hospital GVM Care&Research, Bari; Sapienza University of Rome-Policlinico Umberto I, Rome; “SS Antonio e Biagio e Cesare Arrigo” Hospital, Alessandria; Maria Pia Hospital, Turin; San Giovanni di Dio e Ruggi d’Aragona, Salerno; Ospedale di Lecco, Lecco; Azienda Ospedaliera Universitaria di Parma, Parma; Policlinico Universitario Mater Domini, Catanzaro; IRCCS San Martino, Genova; Ospedale Bolognini, Seriate; Ospedale Civile di Legnano, Legnano; Ospedale S. Chiara, Trento; Istituto Clinico San Rocco, Ome; Fondazione Poliambulanza, Brescia; Maria Cecilia Hospital, Cotignola; European Hospital, Rome (all in Italy). Written informed consent was obtained from all subjects. Additional details are also available in the online registration module on clinicaltrials.gov (NCT02713932).

### Aims

The main aim of this RISPEVA subanalysis was to compare patients undergoing attempted implantation of Acurate, Evolut, Lotus, Portico, or Sapien3 devices, focusing on short-term (procedural, peri-procedural, and 1-month) outcomes. Labelling was based on attempt to deliver and/or deploy a specific device, and not necessarily on eventually successful implantation.

### Patients, procedures, definitions

Details on patient and procedures in the RISPEVA Study have also been detailed previously. Briefly, all patients in whom TAVI was attempted at participating centers and willing to provide consent were offered inclusion in the study, without any additional selection criterion. Several baseline, procedural and outcome variables were collected in a dedicated electronic case report form. Procedural outcomes included: contrast volume, fluoroscopy time, procedural time, device success, procedural success, death, valve migration, anulus rupture, surgical conversion, coronary occlusion, myocardial infarction, pericardial tamponade, aortic dissection, major vascular complication, and major bleeding. Follow-up assessments were planned at 1 month after TAVI and subsequently. In particular, details on the following clinically relevant outcomes were systematically collected: death, cardiac death, surgical aortic valve replacement, valve thrombosis, valve degeneration, endocarditis, coronary occlusion, myocardial infarction, pericardial effusion, stroke, transient ischemic attack, major vascular complication, amputation, major bleeeding, renal failure, and PPI. Echocardiographic assessment was routinely performed at admission, at discharge, and at 1-month, focusing on the following endpoints: veft ventricular end-diastolic diameter (LVEDD), left ventricular end-systolic diameter (LVESD), left ventricular ejection fraction (LEVF), peak aortic gradient, mean aortic gradient, aortic valve area, aortic regurgitation, mitral regurgitation, and systolic pulmonary artery pressure (SPAP). Major adverse events, a composite of death, stroke, myocardial infarction, major vascular complication, major bleeding, or renal failure, were also computed. All definitions originated by the current Valve Academic Research Consortium recommendations^13^.

### Statistical analysis

Continous variables are reported as mean ± standard deviation, and categorical variables as count (%). Unadjusted analysis was based on analysis of variance for continuous variables and chi^2^ test for categorical variables. Adjusted analysis was based on propensity score adjusted generalized linear models with default link for continuous variables and binomial likelihood for binary variables, using missing data imputation when appropriate. Pairwise propensity scores were generated using several baseline and procedural variables (Online supplement). Statistical significance for hypothesis testing was be set at the two-tailed 0.05 level, without multiplicity adjustment. Computations were performed with Stata 13 (StataCorp, College Station, TX).

## Results

### Baseline features

A total of 1976 patients were included, who underwent TAVI between 2012 and 2018, mostly in 7 high volume centers (Table S1): 234 (11.8%) treated with Acurate, 703 (35.6%) with Evolut, 151 (7.6%) with Lotus, 347 (17.6%) with Portico, and 541 (27.4%) with Sapien3. Baseline features are provided in Table 1, with additional details in Table S1. Several differences in baseline features, with complex patterns in favor or against a given device, were found (Fig. S1). Specifically, there were significant differences in age, gender (with more females receiving Acurate and Portico), height, BMI, diagnosis, risk (with more inoperable patients receiving Evolut), Logistic EuroSCORE (with lower values in those receiving Acurate), EuroSCORE II, and STS score (all p < 0.05). Similarly, significant differences were found in NYHA class, prior valvuloplasty, peak aortic gradient, aortic valve area, aortic regurgitation, porcelain aorta, family history of CAD, hypertension, current smoking, prior CAD, angiographic CAD, prior acute pulmonary edema, prior myocardial infarction, prior percutaneous coronary intervention, syncope, carotid artery disease, peripheral artery disease, systolic blood pressure, diastolic blood pressure, hematocrit, oxygen dependency, prior cancer, LVEDV, LVESV, bicuspid aortic valve, and ilio-femoral tortuosity (all p < 0.05).

**Table 1:** Baseline features at unadjusted analysis.

Feature	Acurate	Evolut	Lotus	Portico	Sapien3	P
Patients	234	703	151	347	541	—
Age (years)	83.5 ± 6.0	82.1 ± 6.7	82.0 ± 6.5	82.5 ± 6.5	83.1 ± 6.5	0.015
Female gender	156 (66.7%)	391 (55.6%)	83 (55.0%)	223 (64.3%)	288 (53.2%)	<0.001
Body mass index (kg/m^2^)	26.1 ± 4.5	26.1 ± 4.4	25.4 ± 3.9	26.8 ± 4.6	26.1 ± 4.6	0.045
Diagnosis						<0.001
Aortic stenosis	217 (92.7%)	593 (85.4%)	129 (85.4%)	291 (83.9%)	472 (87.3%)	
Mixed aortic valve disease	9 (3.9%)	76 (10.8%)	18 (11.9%)	15 (4.3%)	53 (9.8%)	
Aortic regurgitation	5 (2.1%)	17 (2.4%)	1 (0.7%)	1 (0.3%)	2 (0.4%)	
Degenerated bioprosthesis	3 (1.3%)	17 (2.4%)	3 (2.0%)	40 (11.5%)	14 (2.6%)	
Risk						<0.001
Inoperable	5 (2.1%)	45 (6.4%)	5 (3.3%)	6 (1.7%)	50 (9.2%)	
High	195 (83.3%)	618 (87.9%)	141 (93.4%)	278 (80.1%)	433 (80.0%)	
Intermediate	34 (14.5%)	40 (5.7%)	5 (3.3%)	63 (18.2%)	58 (10.7%)	
Logistic EuroSCORE	12.9 ± 11.0	16.7 ± 11.6	16.3 ± 14.5	16.2 ± 11.6	16.7 ± 12.1	<0.001
EuroSCORE II	4.1 ± 4.2	5.0 ± 4.9	5.0 ± 6.1	4.2 ± 3.9	5.5 ± 4.8	<0.001
STS score	5.0 ± 3.8	5.6 ± 4.1	5.1 ± 4.2	6.3 ± 4.2	5.4 ± 4.2	0.054
New York Heart Association class						<0.001
I	1 (0.4%)	6 (0.9%)	5 (3.3%)	9 (2.6%)	13 (2.4%)	
II	70 (30.0%)	210 (31.8%)	35 (23.2%)	80 (23.1%)	187 (34.6%)	
III	159 (68.2%)	407 (61.6%)	104 (68.9%)	251 (72.3%)	323 (59.7%)	
IV	3 (1.3%)	38 (5.8%)	7 (4.6%)	7 (2.0%)	18 (3.3%)	
Prior cardiac surgery	21 (9.0%)	38 (5.4%)	13 (8.6%)	22 (6.3%)	40 (7.4%)	0.276
Prior aortic valvuloplasty	12 (5.1%)	33 (4.7%)	13 (8.6%)	14 (4.0%)	52 (9.6%)	0.001
Pacemaker dependency	25 (10.7%)	50 (7.1%)	13 (8.6%)	31 (8.9%)	39 (7.2%)	0.407
Prior stroke or transient ischemic attack	21 (9.0%)	38 (5.4%)	13 (8.6%)	22 (6.3%)	40 (7.4%)	0.276
Estimated glomerular filtration rate (mL/min)	65.1 ± 23.0	62.2 ± 23.8	66.5 ± 47.7	61.1 ± 24.8	62.6 ± 23.6	0.217
Left ventricular ejection fraction (%)	53 ± 11	52 ± 10	53 ± 12	54 ± 10	53 ± 10	0.424
Peak aortic gradient (mm Hg)	75.4 ± 21.3	76.4 ± 22.9	77.7 ± 23.4	71.4 ± 23.6	77.7 ± 20.9	0.014
Mean aortic gradient (mm Hg)	47.8 ± 13.8	47.3 ± 14.9	48.0 ± 14.8	48.0 ± 16.8	48.2 ± 13.8	0.896
Aortic valve area (cm2)	0.67 ± 0.23	0.67 ± 0.26	0.66 ± 0.24	0.69 ± 0.24	0.63 ± 0.18	0.040
Aortic regurgitation						<0.001
None	89 (38.0%)	204 (29.0%)	45 (29.8%)	140 (40.4%)	124 (22.9%)	
1+	96 (41.0%)	320 (45.5%)	71 (47.0%)	123 (35.5%)	302 (55.8%)	
2+	36 (15.4%)	136 (19.4%)	29 (19.2%)	68 (19.6%)	103 (19.0%)	
3+	13 (5.6%)	43 (6.1%)	6 (4.0%)	16 (4.6%)	12 (2.2%)	
Porcelain aorta	8 (3.4%)	58 (8.3%)	9 (6.0%)	4 (1.2%)	49 (9.1%)	<0.001
Angiographically significant coronary artery disease	62 (26.5%)	198 (28.2%)	34 (22.5%)	44 (12.7%)	140 (25.9%)	<0.001
Iliofemoral tortuosity						<0.001
Mild	73 (64.0%)	121 (33.8%)	41 (49.4%)	100 (40.2%)	135 (47.2%)	
Moderate	39 (34.2%)	193 (53.9%)	40 (48.2%)	145 (58.2%)	127 (44.4%)	
Severe	2 (1.8%)	44 (12.3%)	2 (2.4%)	4 (1.6%)	24 (8.4%)	

### Procedural features

Procedural characteristics were also different in many domains (Table 2), including local anesthesia, transesophageal guidance, femoral access, sheathless procedure, sheath size, embolic protection device, right ventricular pacing, predilation, predilation balloon diameter, predilation balloon type, prostheses number, device size, pacing during implant, pacing rate, postdilation, postdilation balloon diameter, and postdilation balloon diameter (all p < 0.05). In particular, transesophageal guidance was less common with Evolut and Sapien3, smaller balloons were used with Lotus, pacing was more common with Evolut and Sapien3, and postdilation less prevalent with Lotus and Sapien3 (all p < 0.05). Despite such differences, procedural and device success were both very high, ranging between 98% and 99%, and non-significantly different across devices. Similarly, unadjusted procedural rates of death, anulus rupture, surgical conversion, coronary occlusion, myocardial infarction, pericardial tamponade, aortic dissection, or major vascular complications were all similar (all p > 0.05), whereas valve migration was more common with Acurate, and major bleeding with Sapien3 (both p < 0.05).

**Table 2:** Procedural features at unadjusted analysis.

Feature	Acurate	Evolut	Lotus	Portico	Sapien3	P
Patients	234	703	151	347	541	—
Local anesthesia	202 (86.3%)	594 (84.5%)	134 (88.7%)	313 (90.2%)	420 (77.6%)	<0.001
Transephageal guidance	76 (32.5%)	10 (1.4%)	47 (31.1%)	128 (36.9%)	51 (9.4%)	<0.001
Femoral access	216 (92.3%)	612 (87.1%)	141 (93.4%)	303 (87.3%)	510 (94.3%)	<0.001
Percutaneous approach	208 (88.9%)	616 (87.6%)	130 (86.1%)	305 (87.9%)	464 (85.8%)	0.732
Sheathless procedure	5 (2.1%)	160 (22.8%)	4 (2.7%)	6 (1.7%)	26 (4.8%)	<0.001
Sheath size (French)	18.4 ± 2.1	15.1 ± 1.7	18.4 ± 1.8	18.4 ± 0.7	14.5 ± 1.7	<0.001
Embolic protection device						<0.001
None	232 (99.2%)	702 (99.9%)	150 (99.3%)	345 (99.4%)	519 (95.9%)	
Claret	1 (0.4%)	1 (0.1%)	1 (0.7%)	2 (0.6%)	20 (3.7%)	
Shimon	1 (0.4%)	0	0	0	2 (0.4%)	
Right ventricular pacing	118 (50.4%)	422 (60.0%)	60 (39.7%)	192 (55.3%)	452 (83.6%)	<0.001
Predilation						<0.001
None	82 (35.0%)	272 (38.7%)	93 (61.6%)	117 (33.7%)	92 (17.0%)	
One balloon	149 (63.7%)	417 (59.3%)	58 (38.4%)	225 (64.8%)	449 (83.0%)	
Two balloons	3 (1.3%)	14 (2.0%)	0	5 (1.4%)	0	
Balloon diameter (mm)	21.8 ± 2.9	20.4 ± 2.0	20.3 ± 1.9	20.7 ± 1.6	21.6 ± 2.0	<0.001
Balloon type						<0.001
Cristal	52 (34.2%)	19 (4.4%)	0	25 (10.9%)	7 (1.6%)	
Nucleus	3 (2.0%)	37 (8.5%)	7 (12.1%)	0	4 (0.9%)	
Z.Med	24 (15.8%)	72 (16.6%)	23 (39.7%)	1 (0.4%)	4 (0.9%)	
Other	73 (48.0%)	307 (70.6%)	28 (48.3%)	204 (88.7%)	434 (96.7%)	
Prosthesis						0.029
One	230 (98.3%)	681 (96.9%)	150 (99.3%)	340 (98.0%)	537 (99.3%)	
Two	4 (1.7%)	22 (3.1%)	1 (0.7%)	7 (2.0%)	4 (0.7%)	
Heterogenous device	1 (0.4%)	0	0	0	0	0.114
Device size (French)	25.0 ± 2.1	28.1 ± 3.1	24.9 ± 2.0	26.4 ± 2.2	25.0 ± 2.4	<0.001
Pacing during implant	71 (30.3%)	152 (21.6%)	16 (10.6%)	18 (5.2%)	501 (92.6%)	<0.001
Pacing rate (bpm)	181 ± 25	158 ± 36	145 ± 59	163 ± 24	182 ± 12	<0.001
Postdilation	111 (47.4%)	202 (28.7%)	2 (1.3%)	165 (47.6%)	27 (5.0%)	<0.001
Balloon diameter (mm)	23.2 ± 1.9	24.1 ± 2.6	20 ± 0	23.9 ± 2.0	22.4 ± 2.1	<0.001
Balloon length (mm)	42.1 ± 4.7	40.8 ± 2.4	40.0 ± 0	41.1 ± 2.9	40.9 ± 7.4	0.415
Fluoroscopy time (seconds)	22.7 ± 13.5	26.3 ± 16.3	30.0 ± 11.0	26.4 ± 14.1	21.5 ± 14.8	<0.001
Procedural time (minutes)	120.5 ± 50.8	113.0 ± 51.5	104.5 ± 39.6	87.4 ± 43.1	113.1 ± 46.6	<0.001
Device success	232 (99.2%)	693 (98.6%)	148 (98.0%)	343 (98.9%)	533 (98.5%)	0.899
Procedural success	232 (99.2%)	693 (98.6%)	148 (99.4%)	345 (99.4%)	533 (98.5%)	0.627
Complications						
Death	1 (0.4%)	2 (0.3%)	3 (2.0%)	1 (0.3%)	6 (1.1%)	0.085
Valve migration	5 (2.1%)	7 (1.0%)	0	0	1 (0.2%)	0.007
Anulus rupture	0	0	0	0	2 (0.4%)	0.257
Surgical conversion	0	1 (0.1%)	1 (0.7%)	2 (0.6%)	2 (0.4%)	0.576
Coronary occlusion	0	1 (0.1%)	0	0	2 (0.4%)	0.590
Myocardial infarction	0	1 (0.1%)	0	0	1 (0.2%)	0.873
Pericardial tamponade	1 (0.4%)	2 (0.3%)	3 (2.0%)	2 (0.6%)	8 (1.5%)	0.069
Aortic dissection	0	3 (0.4%)	0	3 (0.9%)	2 (0.4%)	0.492
Major vascular complication	7 (3.0%)	25 (3.6%)	5 (3.3%)	11 (3.2%)	21 (3.9%)	0.969
Major bleeding	8 (3.4%)	39 (5.6%)	5 (3.3%)	8 (2.3%)	43 (8.0%)	0.002

### Unadjusted analysis for one-month outcomes

Most one-month outcomes were similar across devices (Table 3), including death, cardiac death, surgical aortic valve replacement, valve thrombosis, valve degeneration, endocarditis, coronary occlusion, myocardial infarction, stroke, transient ischemic attack, amputation, and major bleeding (all p > 0.05). However, significant differences were found for major adverse events (p < 0.001, with lowest rates for Portico), pericardial effusion (p < 0.001, with lowest rates for Portico), major vascular complication (p < 0.001, with lowest rates for Portico), and PPI (p < 0.001, with lowest rates for Acurate). Echocardiographic follow-up showed differences in LVEDD, peak aortic gradient, mean aortic gradient, aortic valve area, aortic regurgitation, and mitral regurgitation (all p < 0.05).

**Table 3:** One-month outcomes at unadjusted analysis.

Feature	Acurate	Evolut	Lotus	Portico	Sapien3	P
Patients	234	703	151	347	541	—
Clinical outcomes						
Major adverse event*	60 (25.6%)	135 (19.2%)	24 (15.9%)	40 (11.5%)	131 (24.2%)	<0.001
Death	4 (1.7%)	11 (1.6%)	4 (2.7%)	8 (2.3%)	8 (1.5%)	0.794
Cardiac death	1 (0.4%)	4 (0.6%)	3 (2.0%)	2 (0.6%)	6 (1.1%)	0.357
Surgical aortic valve replacement	0	1 (0.1%)	1 (0.7%)	3 (0.9%)	2 (0.4%)	0.320
Valve thrombosis	0	2 (0.3%)	0	0	1 (0.2%)	0.742
Valve degeneration	0	0	0	0	0	1
Endocarditis	0	0	0	0	0	1
Coronary occlusion	0	1 (0.1%)	0	0	2 (0.4%)	0.590
Myocardial infarction	0	2 (0.3%)	0	0	2 (0.4%)	0.650
Pericardial effusion	12 (5.1%)	27 (3.8%)	6 (4.0%)	8 (2.3%)	46 (8.5%)	<0.001
Stroke	1 (0.4%)	12 (1.7%)	4 (2.7%)	2 (0.6%)	7 (1.3%)	0.216
Transient ischemic attack	0	2 (0.3%)	0	1 (0.3%)	0	0.618
Stroke or transient ischemic attack	1 (0.4%)	13 (1.9%)	4 (2.7%)	3 (0.9%)	7 (1.3%)	0.283
Major vascular complication	33 (14.1%)	61 (8.7%)	8 (5.3%)	17 (4.9%)	68 (12.6%)	<0.001
Amputation	0	0	0	0	0	1
Major bleeeding	10 (4.3%)	20 (2.8%)	7 (4.6%)	9 (2.6%)	27 (5.0%)	0.218
Renal failure	26 (11.1%)	63 (9.0%)	9 (6.0%)	16 (4.6%)	47 (8.7%)	0.035
Permanent pacemaker	13 (5.6%)	122 (17.4%)	35 (23.2%)	42 (12.1%)	72 (13.3%)	<0.001
Echocardiographic outcomes						
Left ventricular end-diastolic diameter (mm)	45.6 ± 9.3	49.2 ± 10.5	49.6 ± 8.1	46.7 ± 6.9	49.1 ± 8.5	0.002
Left ventricular end-systolic diameter (mm)	32.1 ± 8.2	32.3 ± 10.5	33.2 ± 9.5	31.6 ± 8.0	32.1 ± 11.5	0.971
Left ventricular ejection fraction (%)	55.2 ± 9.5	53.3 ± 9.6	52.9 ± 10.4	54.2 ± 9.1	53.2 ± 9.2	0.051
Peak aortic gradient (mm Hg)	16.0 ± 9.5	15.3 ± 7.9	19.6 ± 14.0	18.2 ± 13.2	21.6 ± 11.5	<0.001
Mean aortic gradient (mm Hg)	9.2 ± 0.4.4	8.2 ± 4.6	12.1 ± 5.4	9.4 ± 6.1	11.6 ± 5.1	<0.001
Aortic valve area (mm2)	1.17 ± 0.47	1.43 ± 0.49	1.28 ± 0.37	1.04 ± 0.47	1.37 ± 0.47	<0.001
Aortic regurgitation						<0.001
None	44 (20.9%)	207 (35.3%)	91 (67.4%)	133 (44.5%)	240 (50.0%)	
1+	135 (64.0%)	309 (52.7%)	40 (29.6%)	139 (46.5%)	224 (46.7%)	
2+	32 (15.2%)	66 (11.3%)	4 (3.0%)	26 (8.7%)	16 (3.3%)	
3+	0	4 (0.7%)	0	1 (0.3%)	0	
4+	0	0	0	0	0	
Mitral regurgitation						<0.001
None	12 (5.2%)	42 (7.0%)	6 (4.2%)	20 (6.0%)	21 (4.0%)	
1+	131 (57.2%)	367 (61.0%)	87 (60.4%)	199 (59.8%)	263 (50.3%)	
2+	64 (28.0%)	37 (6.2%)	42 (29.2%)	86 (25.8%)	193 (36.9%)	
3+	15 (6.6%)	140 (23.3%)	8 (5.6%)	20 (6.0%)	36 (6.9%)	
4+	7 (3.1%)	16 (2.7%)	1 (0.7%)	8 (2.4%)	10 (1.9%)	
Systolic pulmonary artery pressure (mm Hg)	36.7 ± 11.8	38.1 ± 15.0	38.2 ± 11.9	37.8 ± 11.0	35.5 ± 9.5	0.052

^*^Composite of death, stroke, myocardial infarction, major vascular complication, major bleeding, or renal failure.

### Adjusted analysis

**Figure 1:**
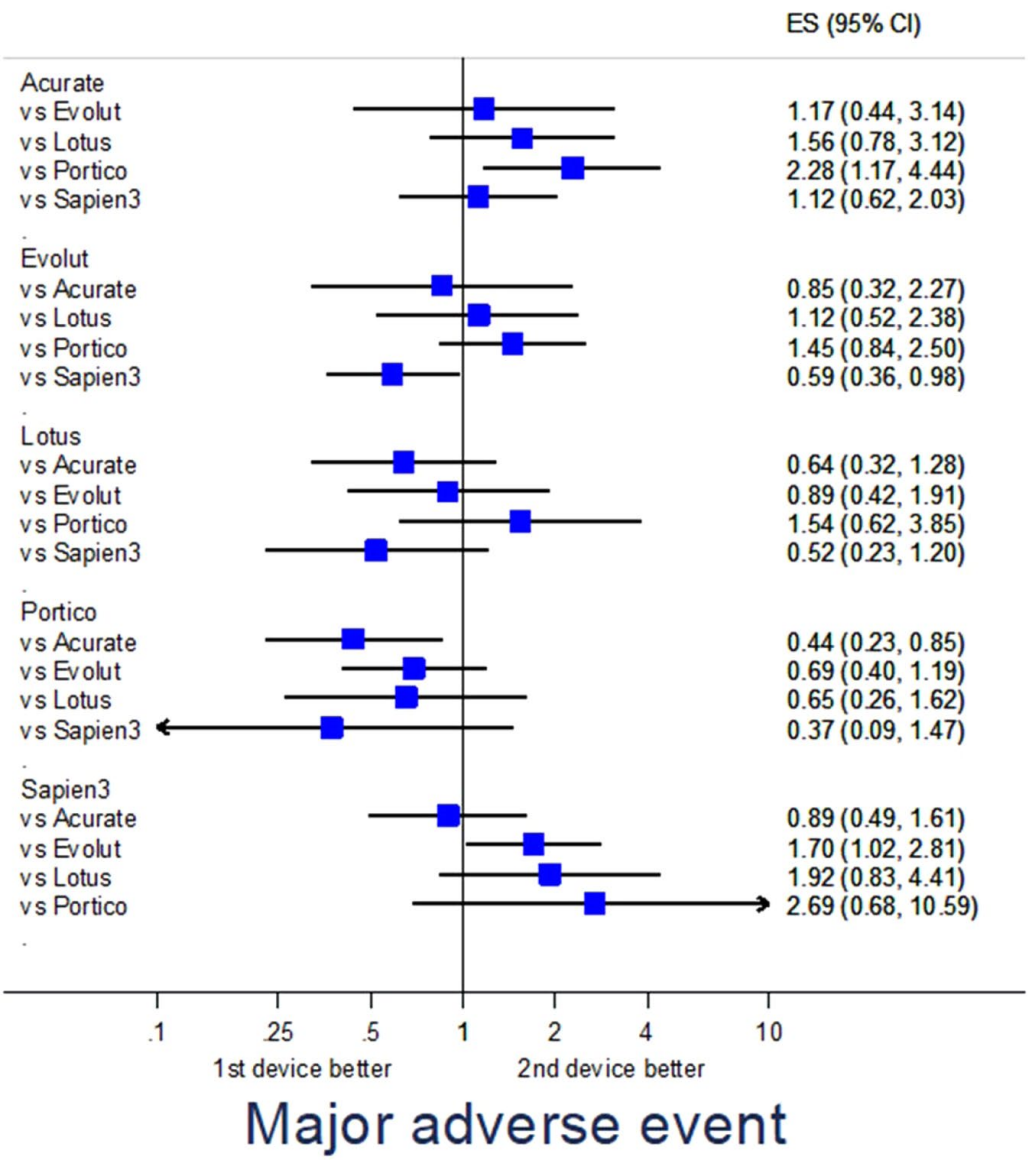
Forest plot of propensity score-adjusted analysis for major adverse events (composite of death, stroke, myocardial infarction, major vascular complication, major bleeding, or renal failure).

**Figure 2:**
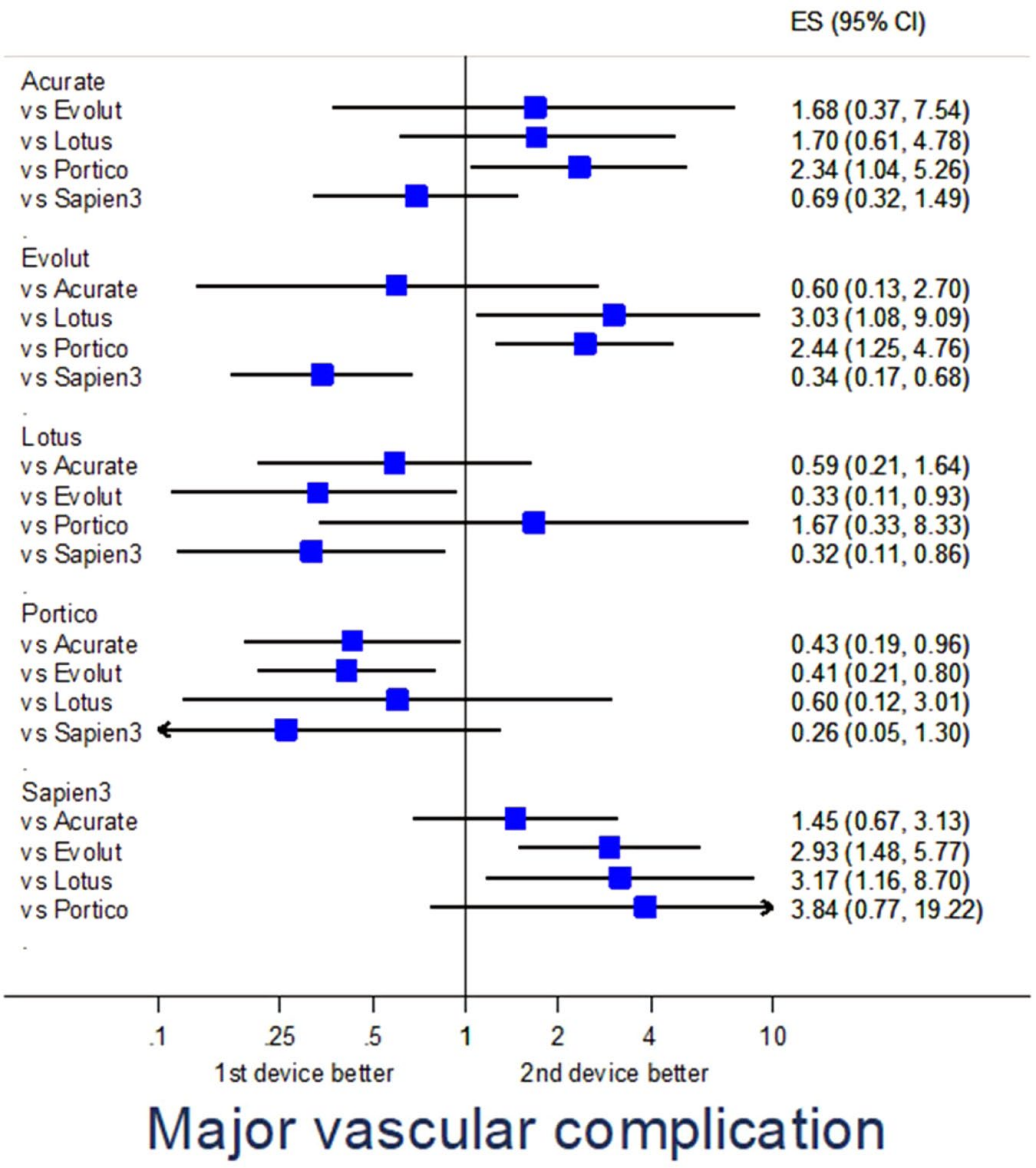
Forest plot of propensity score-adjusted analysis for major vascular complication.

**Figure 3:**
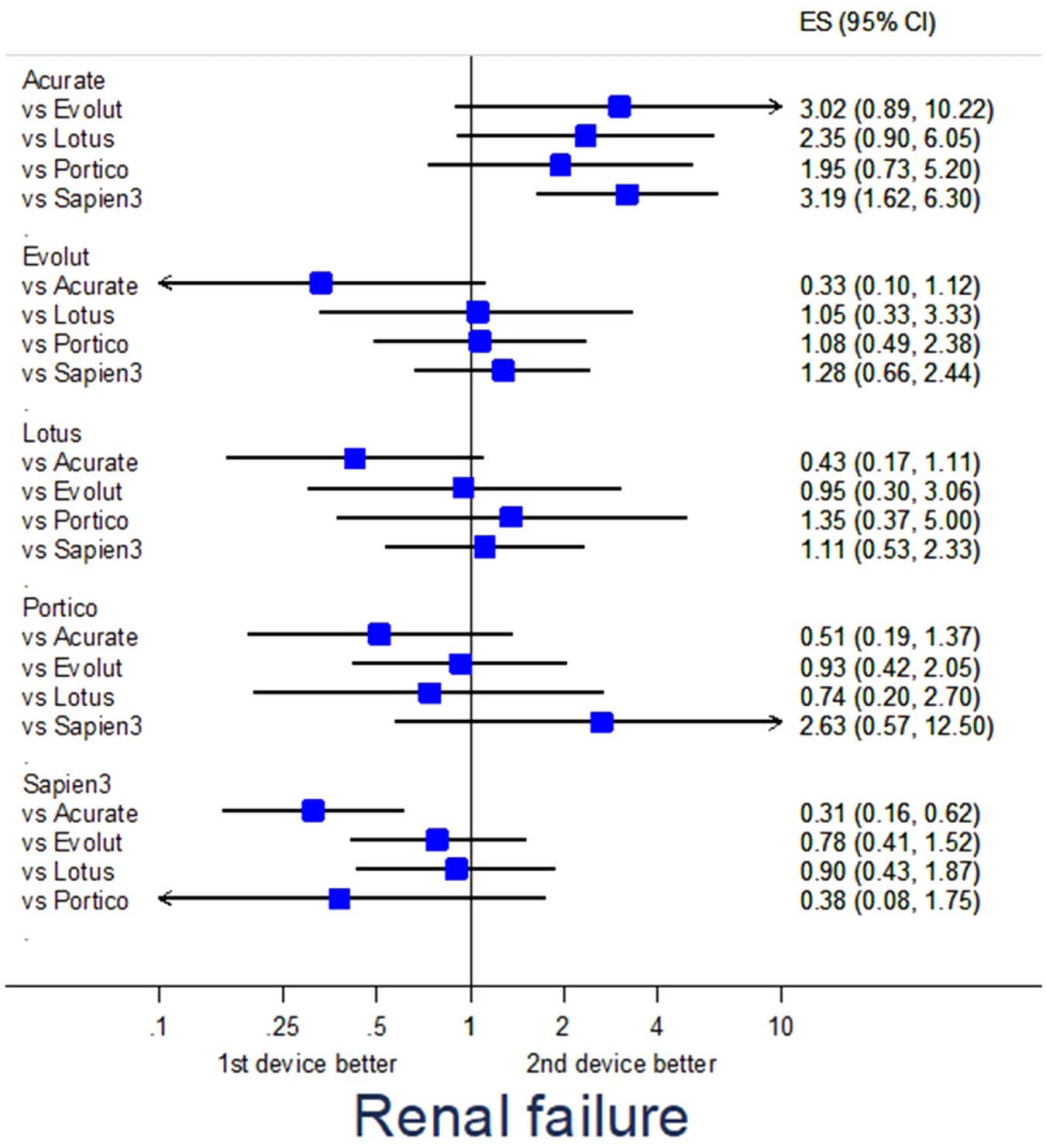
Forest plot of propensity score-adjusted analysis for renal failure.

**Figure 4:**
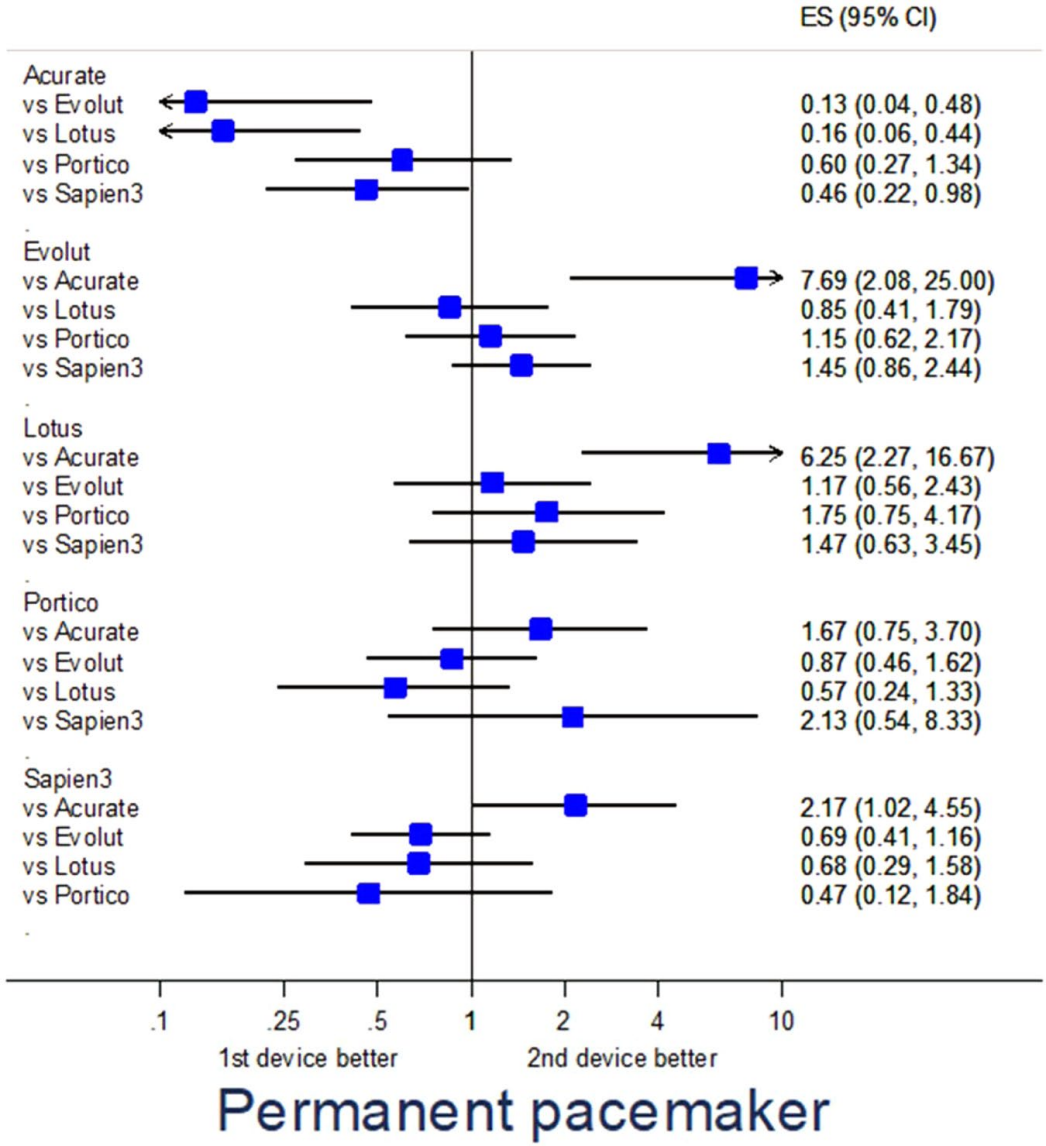
Forest plot of propensity score-adjusted analysis for permanent pacemaker implantation.

Propensity score-adjusted analysis for selected endpoints is provided in Table S3. Specifically, major adverse events were fewest with Evolut (p = 0.040 vs Sapien3) and Portico (p = 0.016 vs Acurate) (Fig. 1), major vascular complications were less common with Evolut (p = 0.036 vs Lotus, p = 0.002 vs Sapien3), Lotus (p = 0.024 vs Sapien3), and Portico (p = 0.040 vs Acurate, p = 0.010 vs Evolut) (Fig. 2), renal failure was less frequent with Sapien3 (p = 0.001 vs Acurate) (Fig. 3), and PPI was less common with Acurate (p = 0.002 vs Evolut, p < 0.001 vs Lotus, p = 0.043 vs Sapien3) (Fig. 4).

**Figure 5:**
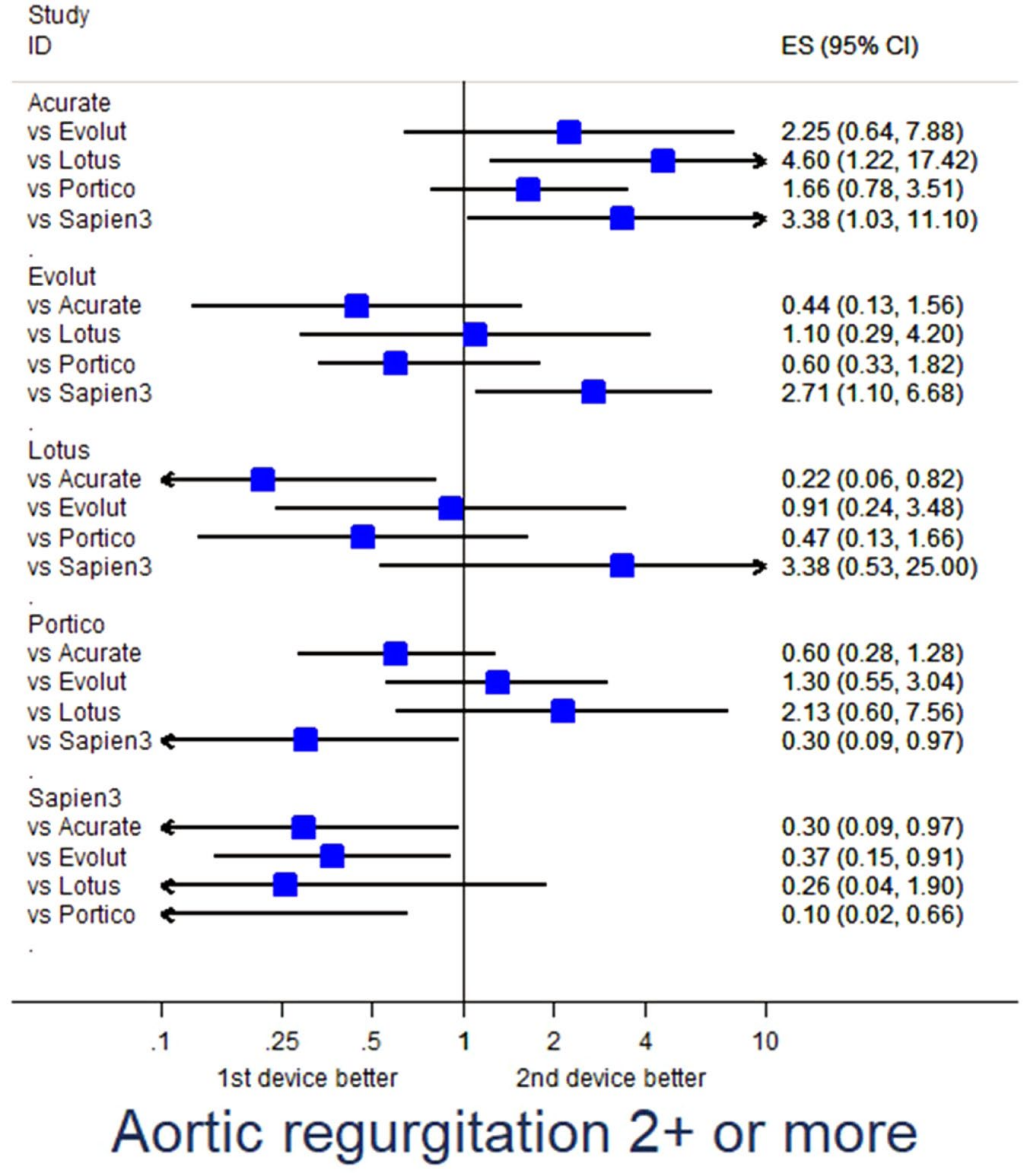
Forest plot of propensity score-adjusted analysis for aortic regurgitation implantation ≥2+.

Adjusted analysis for echocardiographic features suggested lower mean aortic gradients with Acurate (p = 0.002 vs Lotus, p = 0.023 vs Sapien3), Evolut (p < 0.001 vs Lotus, p < 0.001 vs Sapien3), and Portico (p = 0.033 vs Lotus, p = 0.043 vs Sapien3), less aortic regurgitation ≥ 2 + /4+ with Sapien3 (p = 0.045 vs Acurate, p = 0.030 vs Evolut, p = 0.017 vs Portico) and Lotus (p = 0.025 vs Acurate), and less mitral regurgitation with Evolut (p = 0.017 vs Portico, p < 0.001 vs Sapien3) (Fig. 5).

### Additional analysis

For exploratory purposes, we also appraised the impact of overall TAVI experience per center, as well as device-specific experience (Tables S4; S5). At unadjusted analysis using tertiles, instituions at high overall volume showed lower rates of major adverse events, death, and myocardial infarction than other centers (all p < 0.05), whereas PPI appeared less common in low volume institutions (p = 0.009). Device-specific experience did not seemed associated with significant differences in outcomes, with the notable exception of the risk of death with Lotus, which appeared higher in institutions less experienced with this device (p = 0.010).

Additional sensitivity inferential analyses were conducted forcing in the propensity score-adjusted model overall and device-specific experience (Table S6). These analyses, albeit limited by multiplicity issues, suggested that in more experienced settings Portico outperformed Acurate (p = 0.012) and Evolut proved better than Sapien3 (p = 0.045). Focusing on device-specific institutional experience, Portico proved better than Evolut in centers experienced with the latter device (p = 0.015), whereas in the same setting Evolut proved nonetheless better than Sapien3 (p = 0.025).

## Discussion

### Main findings

This analysis of the RISPEVA study focusing on short-term outcomes following TAVI with 5 leading new-generation devices, despite its mainly descriptive and hypothesis-generating scope, has the following implications: (a) centers and operators performing TAVI used different devices based on preference, training, experience, and patient features; (b) despite substantial differences in baseline and procedural features, ranging from age and gender to subtleties such as pacing or transesophageal guidance, rates of device and procedural success, as well as those of several procedural outcomes including death, appeared largely similar with Acurate, Evolut, Lotus, Portico and Sapien3; (c) accordingly, one-month outcomes appeared quite similar across devices, with encouraging results in terms of death (<3% for all devices), as well as myocardial infarction, stroke and major bleeding; (d) differences were however apparent across devices for major vascular complications, renal failure and PPI; (e) propensity score-adjusted analysis confirmed such trends, highlighting that these devices may not be considered completely equivalent, and individualized decision making and device choice probably remain important to maximize the risk-benefit and cost-benefit profile of TAVI.

The present findings appear important and timely, and supplement recent ones by other investigators who aimed at comparing different TAVI devices^1^. Overall, all recent reports reaffirm the safety of TAVI, confirming its established role in patients with aortic stenosis at intermediate to prohibitive surgical risk, and sustaining recent trials testing the role of TAVI in low risk patients^2,3^. Indeed, major adverse events were quite uncommon, despite this being a pragmatic registry without any specific selection or exclusion criteria. Yet, uncertainty persists on the long-term durability of TAVI, especially in light of recent reports on valve deterioration and leaflet thrombosis (albeit typically silent)^4–6^. Indeed, structural valve degeneration occurred in 8.7% of patients receiving first-generation TAVI devices after a median follow-up of 5.8 years in the UK TAVI Registry, similar to the 4.8% estimate provided after 6 years of follow-up in the Nordic Aortic Valve Intervention (NOTION) trial^14,15^. Accordingly, much emphasis must be given to the long-term comparative safety and efficacy of TAVI, especially when considering lower risk patients as potential candidates. Despite the ever accruing evidence base, operators are faced with novel devices or refinements of existing ones, with uncertainty on their incremental differences.

### Context

Balloon-expandable devices have been originally considered superior to self-expandable devices in terms of PPI and aortic regurgitation rates, but were associated with higher rates of strokes and vascular complications, at least in some series or comparisons^16,17^. More recent data support the existence of meaningful differences between Evolut and Sapien, between Evolut and Portico, and possibly between Lotus and Sapien3^9,12^. Furthermore, the temporary recall of Lotus depended on engineering issues likely of clinical impact, despite this device usefulness in minimizing aortic regurgitation, albeit at the potential expense of an increase in PPI rates. Other notices of caution or recalls have been recently issued for Sapien3. Thus, attentive focus on each device strenths and weaknesses is paramount. Indeed, in light of prior and recent comparative studies on new-generation TAVI devices as well as our own present ones, we may first infer that experienced operators who have confidence with a given device can obtain satisfactory results with any chosen product, but probably each institution should consider becoming familiar with at least two devices with different features, in order to maximize the benefit of individualized device choice, without compromising skills. Indeed, in keeping with our head-to-head comparisons, it appears that Portico and Evolut are associated with lower rates of major adverse events (p = 0.016 in favor of Portico when compared to Acurate; p = 0.040 in favor of Evolut when compared to Sapien3), Portico with fewer vascular complications (p < 0.05 in its favor when compared to Acurate and Evolut), Sapien3 with fewer renal failures (p = 0.001 in its favor when compared to Acurate), and Acurate with lower PPI rates (p < 0.05 in its favor when compared to Evolut, Lotus, and Sapien3). Echocardiographic analyses showed lower gradients with Acurate, Evolut and Portico, with aortic regurgitation and paravalvular leak appearing less common with Lotus and Sapien3. Notably, mean echocardiographic gradients appeared superior in self-expandable valves than in mechanically or balloon-expandable valves irrespective of valve-anulus-height, with potentially detrimental impact on long-term durability.

Focusing on institutional experience with TAVI in general and with specific devices, we found intriguing hypothesis-generating data, suggesting that clinical outcomes are better in higher volume centers, except for PPI, whose rates may indeed depend on local management protocols. Focusing on specific devices, only Lotus seemed significantly dependant on experience. Furthermore, we found that overall and device-specific experience did not dilute significant differences between devices, with persistent evidence that Portico outperformed Acurate and Evolut, with the latter still having an edge in comparison to Sapien3. Yet, these findings, while intriguing, need confirmation in long-term follow-up from RISPEVA and, most importantly, from ongoing comparative randomized trials^1^.

### Limitations

On top of being limited by the non-randomized design, short-term follow-up, and reliance on propensity score adjustment, this study is limited by incomplete data collection for several ancillary baseline features such as frailty, absence of an imaging core lab, and lack of systematic endpoint adjudication. In addition, very few cases of TAVI with other devices such as JenaValve or Allegra were collected, and these devices were thus excluded from analysis. Notably, residual confounding cannot be excluded and might drive some important differences, even after propensity score adjustment, such as for vascular complications. In addition, while we attempted at exploring the impact of institutional experience with TAVI in general and with specific devices as well, operator volume and experience was not collected in the case report form, and thus could not be analyzed.

## Conclusion

New-generation TAVI devices have different profiles of early comparative safety and efficacy. These findings should be taken into account for individualized decision making and patient management.

## Supplementary information

Supplementary Information

## Data Availability

Anonymized data are available for external analysis from the corresponding author upon request.
