# Supplementary material for: Comparative one-month safety and effectiveness of five leading new-generation devices for transcatheter aortic valve implantation
Source: Sci Rep. 2019 Nov 19;9:17098. doi: 10.1038/s41598-019-53081-w (PMC6864033; doi:10.1038/s41598-019-53081-w)

**Online supplement to:**

**Comparative one-month safety and effectiveness of five leading new-generation devices for transcatheter aortic valve implantation**

Arturo Giordano, MD, PhD,^1^ Nicola Corcione, MD,^1^ Paolo Ferraro, MD,^2^ Alberto Morello, MD,^1^ Sirio Conte, MD,^2^ Luca Testa, MD,^3^ Francesco Bedogni, MD,^3^ Alessandro Iadanza, MD,^4^ Sergio Berti, MD,^5^ Damiano Regazzoli, MD,^6^ Enrico Romagnoli, MD, PhD,^7^ Carlo Trani, MD,^7^ Francesco Burzotta, MD,^7^ Martino Pepe, MD,^8^ Giacomo Frati, MD, MSc,^9,10^ and Giuseppe Biondi-Zoccai, MD, MStat,^9,11^ on behalf of the Registro Italiano GISE sull'impianto di Valvola Aortica Percutanea (RISPEVA) Study Investigators

^1^Unita` Operativa di Interventistica Cardiovascolare, Pineta Grande Hospital, Castel Volturno, Italy; ^2^Unità Operativa di Emodinamica, Santa Lucia Hospital, San Giuseppe Vesuviano, Italy; ^3^Department of Cardiology, IRCCS Policlinico San Donato, San Donato Milanese, Milan, Italy; ^4^Divisione di Emodinamica, Dipartimento di Scienze Cardiache, Toraciche e Vascolari, Policlinico Santa Maria alle Scotte, Siena, Italy; ^5^Fondazione C.N.R. G. Monasterio Ospedale del Cuore, Massa, Italy; ^6^Department of Biomedical Sciences, Humanitas University, Rozzano, Italy; ^7^Institute of Cardiology, Fondazione Policlinico Universitario A. Gemelli IRCCS, Università Cattolica del Sacro Cuore, Rome, Italy; ^8^Division of Cardiology, Department of Emergency and Organ Transplantation, University of Bari, Bari, Italy; ^9^Department of Medico-Surgical Sciences and Biotechnologies, Sapienza University of Rome, Latina, Italy; ^10^IRCCS NEUROMED, Pozzili, Italy; ^11^Mediterranea Cardiocentro, Napoli, Italy

**List of variables used for propensity score computation**

Device used, age, gender, height, weight, body mass index (BMI), family history of CAD, diabetes mellitus, dyslipidemia, hypertension, current smoking, diagnosis, degenerated bioprosthesis, surgical risk, Logistic EuroSCORE, EuroSCORE II, STS score, New York Heart Association (NYHA) class, prior cardiac surgery, prior aortic valvuloplasty, pacemaker dependency, prior stroke or transient ischemic attack, estimated glomerular filtration rate, LVEF, peak aortic gradient, mean aortic gradient, aortic valve area, aortic regurgitation, porcelain aorta, iliofemoral tortuosity, prior coronary artery disease (CAD), angiographically significant CAD, prior acute pulmonary edema, prior myocardial infarction, prior coronary artery bypass grafting, prior percutaneous coronary intervention, prior carotid intervention, prior iliofemoral intervention, syncope, carotid artery stenosis, peripheral artery disease, systolic blood pressure, diastolic blood pressure, hemoglobin, hematocrit, white blood cells, platelets, cirrhosis, chronic obstructive pulmonary disease, oxygen dependency, cachexia, prior cancer, left ventricular end diastolic volume (LVEDV), left ventricular end systolic volume (LVESV), bicuspid aortic valve, local anesthesia, transephageal guidance, femoral access, percutaneous approach, sheathless procedure, sheath size, embolic protection device, right ventricular pacing, predilation, predilation balloon diameter, predilation balloon type, number of TAVI devices, heterogenous device, device size, pacing during implant, pacing rate, postdilation, postdilation balloon diameter, postdilation balloon length, and imputed dataset

**Table 1S.** List of participating centers enrolling 50 or more patients.

| **Center** | **Investigator(s)** | **City** |
| --- | --- | --- |
| Presidio Ospedaliero Pineta Grande | Arturo Giordano | Castel Volturno |
| Ospedale del Cuore G. Pasquinucci | Sergio Berti | Massa |
| Azienda Ospedaliero-Universitaria Pisana | Anna Sonia Petronio | Pisa |
| Policlinico Agostino Gemelli | Carlo Trani | Roma |
| Istituto Clinico Humanitas | Giulio Stefanini | Rozzano |
| IRCCS Policlinico San Donato | Francesco Bedogni | San Donato Milanese |
| Azienda Ospedaliera Universitaria Senese | Alessandro Iadanza | Siena |

**Table 2S.** Additional baseline features.

| **Feature** | **Acurate** | **Evolut** | **Lotus** | **Portico** | **Sapien3** | **P** |
| --- | --- | --- | --- | --- | --- | --- |
| Patients | 234 | 703 | 151 | 347 | 541 | - |
| Height (cm) | 161.6±8.3 | 163.9±8.6 | 165.2±8.5 | 162.0±7.6 | 164.5±9.0 | <0.001 |
| Weight (kg) | 68.1±12.4 | 70.3±14.1 | 69.3±12.8 | 70.3±13.4 | 70.7±13.9 | 0.185 |
| Family history of coronary artery disease | 28 (12.0%) | 87 (12.4%) | 23 (15.2%) | 16 (4.6%) | 78 (14.4%) | <0.001 |
| Diabetes mellitus |  |  |  |  |  | 0.252 |
| No | 180 (76.9%) | 526 (74.8%) | 116 (76.8%) | 269 (77.5%) | 401 (74.1%) |  |
| Non-insulin-dependent | 39 (16.7%) | 112 (15.9%) | 17 (11.3%) | 58 (16.7%) | 94 (17.4%) |  |
| Insulin-dependent | 15 (6.4%) | 65 (9.3%) | 18 (11.9%) | 20 (5.8%) | 46 (8.5%) |  |
| Dyslipidemia | 123 (52.6%) | 390 (55.5%) | 74 (49.0%) | 169 (48.7%) | 311 (57.5%) | 0.064 |
| Hypertension | 185 (79.1%) | 561 (79.8%) | 118 (78.2%) | 258 (74.4%) | 466 (86.1%) | <0.001 |
| Current smoking | 11 (4.7%) | 57 (8.1%) | 13 (8.6%) | 19 (5.5%) | 63 (11.7%) | 0.003 |
| Prior coronary artery disease | 86 (36.8%) | 242 (34.4%) | 49 (32.5%) | 61 (17.6%) | 175 (32.4%) | <0.001 |
| Prior acute pulmonary edema | 18 (7.7%) | 105 (14.9%) | 19 (13.0%) | 31 (8.9%) | 97 (17.9%) | <0.001 |
| Prior myocardial infarction | 39 (16.7%) | 100 (14.2%) | 17 (11.3%) | 29 (8.4%) | 81 (15.0%) | 0.018 |
| Prior coronary artery bypass grafting | 20 (8.6%) | 46 (6.5%) | 13 (8.6%) | 25 (7.2%) | 48 (8.9%) | 0.574 |
| Prior percutaneous coronary intervention | 59 (25.2%) | 194 (27.6%) | 39 (25.8%) | 76 (21.9%) | 109 (20.2%) | 0.031 |
| Prior carotid intervention | 3 (1.3%) | 21 (3.0%) | 3 (2.0%) | 6 (1.7%) | 20 (3.7%) | 0.229 |
| Prior iliofemoral intervention | 4 (1.7%) | 21 (3.0%) | 2 (1.3%) | 5 (1.4%) | 11 (2.0%) | 0.425 |
| Syncope | 38 (16.2%) | 33 (4.7%) | 18 (11.9%) | 19 (5.5%) | 44 (8.1%) | <0.001 |
| Carotid artery stenosis | 46 (19.7%) | 66 (9.4%) | 29 (19.2%) | 71 (20.5%) | 121 (22.4%) | <0.001 |
| Peripheral artery disease | 25 (10.7%) | 106 (15.1%) | 20 (13.3%) | 24 (6.9%) | 78 (14.4%) | 0.003 |
| Systolic blood pressure (mm Hg) | 138.9±21.4 | 131.8±21.1 | 129.9±17.6 | 129.0±16.3 | 132.4±21.6 | 0.003 |
| Diastolic blood pressure (mm Hg) | 75.0±23.3 | 69.1±12.1 | 81.2±26.4 | 70.9±20.5 | 87.7±31.3 | <0.001 |
| Hemoglobin | 12.3±1.6 | 12.0±1.7 | 12.1±1.7 | 12.2±1.7 | 12.2±1.6 | 0.384 |
| Hematocrit | 37.6±4.6 | 36.7±4.8 | 36.5±4.8 | 36.5±4.8 | 36.4±5.0 | 0.088 |
| White blood cells | 7.1±2.5 | 7.2±2.3 | 7.7±2.9 | 7.1±.1 | 7.0±1.9 | 0.058 |
| Platelets | 212±65 | 210±72 | 212±75 | 211±71 | 203±75 | 0.347 |
| Cirrhosis | 2 (0.9%) | 12 (1.7%) | 1 (0.7%) | 3 (0.9%) | 15 (2.8%) | 0.123 |
| Chronic obstructive pulmonary disease | 33 (14.1%) | 101 (14.4%) | 26 (17.2%) | 55 (15.9%) | 87 (16.1%) | 0.827 |
| Oxygen dependency | 4 (1.7%) | 1 (0.1%) | 3 (2.0%) | 6 (1.7%) | 5 (0.9%) | 0.038 |
| Cachexia | 11 (4.7%) | 21 (3.0%) | 8 (5.3%) | 15 (4.3%) | 14 (2.6%) | 0.284 |
| Prior cancer | 41 (17.5%) | 102 (14.5%) | 21 (13.9%) | 28 (8.1%) | 77 (14.2%) | 0.012 |
| Left ventricular end diastolic volume (mL) | 101±37 | 108±42 | 103±39 | 99±40 | 117±43 | <0.001 |
| Left ventricular end systolic volume (mL) | 47±28 | 56±33 | 50±38 | 46±31 | 65±44 | <0.001 |
| Bicuspid aortic valve |  |  |  |  |  | 0.027 |
| No | 230 (98.3%) | 676 (96.2%) | 146 (96.7%) | 346 (99.7%) | 534 (98.7%) |  |
| Type 0 | 2 (0.9%) | 10 (1.4%) | 4 (2.7%) | 0 | 3 (0.6%) |  |
| Type 1 | 2 (0.9%) | 16 (2.3%) | 1 (0.7%) | 1 (0.3%) | 4 (0.7%) |  |
| Type 2 | 0 | 1 (0.1%) | 0 | 0 | 0 |  |

**Table 3S.** One-month outcomes at propensity score-adjusted analysis.

| **Feature** | **Acurate vs Evolut** | **Acurate vs Lotus** | **Acurate vs Portico** | **Acurate vs Sapien3** | **Evolut vs Lotus** | **Evolut vs Portico** | **Evolut vs Sapien3** | **Lotus vs Portico** | **Lotus vs Sapien3** | **Portico vs Sapien3** |
| --- | --- | --- | --- | --- | --- | --- | --- | --- | --- | --- |
| Clinical outcomes |  |  |  |  |  |  |  |  |  |  |
| Major adverse event* | OR=1.17  (0.44-3.14) P=0.749 | OR=1.56 (0.78-3.12) P=0.206 | OR=2.28 (1.17-4.44) P=0.016 | OR=1.12 (0.62-2.03) P=0.699 | OR=1.12 (0.52-2.38)  P=0.769 | OR=1.45 (0.84-2.50)  P=0.182 | OR=0.59 (0.36-0.98)  P=0.040 | OR=1.54 (0.62-3.85) P=0.355 | OR=0.52 (0.23-1.21)  P=0.125 | OR=0.37 (0.09-1.47) P=0.157 |
| Major vascular complication | OR=1.68  (0.37-7.54)  P=0.498 | OR=1.70 (0.61-4.78) P=0.313 | OR=2.34 (1.04-5.26) P=0.040 | OR=0.69 (0.32-1.49) P=0.348 | OR=0.33 (0.11-0.93)  P=0.036 | OR=2.44 (1.25-4.76)  P=0.010 | OR=0.34 (0.17-0.68)  P=0.002 | OR=1.67 (0.33-8.33) P=0.536 | OR=0.32 (0.12-0.86) P=0.024 | OR=0.26 (0.05-1.30) P=0.100 |
| Renal failure | OR=3.02 (0.89-10.22) P=0.075 | OR=2.35 (0.90-6.05) P=0.080 | OR=1.95 (0.73-5.20) P=0.181 | OR=3.19 (1.62-6.30) P=0.001 | OR=1.05 (0.33-3.33) P=0.934 | OR=1.07 (0.49-2.38) P=0.843 | OR=1.28 (0.66-2.44) P=0.475 | OR=1.35 (0.37-5.00) P=0.650 | OR=1.11 (0.54-2.33) P=0.776 | OR=2.63 (0.57-12.50) P=0.215 |
| Permanent pacemaker | OR=0.13  (0.04-0.48) P=0.002 | OR=0.16 (0.06-0.44) P<0.001 | OR=0.60 (0.27-1.34) P=0.213 | OR=0.46 (0.22-0.98) P=0.043 | OR=0.86 (0.41-1.79) P=0.677 | OR=1.15 (0.62-2.17) P=0.650 | OR=1.45 (0.86-2.44) P=0.161 | OR=1.75 (0.75-4.17) P=0.191 | OR=1.47 (0.63-3.49) P=0.369 | OR=2.13 (0.54-8.33) P=0.274 |
| Echocardiographic outcomes |  |  |  |  |  |  |  |  |  |  |
| Mean aortic gradient (mm Hg) | Β=0.87  (-0.77; 2.52)  P=0.176 | Β=-2.57  (-4.18; -0.95) P=0.002 | Β=-0.54  (-1.67; 0.58) P=0.345 | Β=-1.82  (-3.39; -0.25) P=0.023 | Β=-3.50  (-4.91; -2.09) P<0.001 | Β=-1.20  (-2.64; 0.23) P=0.100 | Β=-4.34  (-5.71; -2.97)  P<0.001 | Β=1.93  (0.16; 3.70)  P=0.033 | Β=-1.28  (-3.29; 0.73) P=0.213 | Β=-3.62  (-7.13; 0.11) P=0.043 |
| Aortic regurgitation ≥2+ | OR=2.25 (0.64-7.88)  P=0.218 | OR=4.60 (1.22-17.42) P=0.025 | OR=1.66 (0.78-3.51) P=0.189 | OR=3.38 (1.03-11.10) P=0.045 | OR=1.10  (0.29-4.20)  P=0.890 | OR=0.60 (0.33-1.82) P=0.547 | OR=2.71  (1.10-6.69)  P=0.030 | OR=0.47 (0.13-1.66)  P=0.240 | OR=3.38 (0.53-25.00) P=0.184 | OR=0.30 (0.09-0.97) P=0.017 |
| Mitral regurgitation ≥2+ | OR=0.58 (0.23-1.48)  P=0.085 | OR=1.46 (0.77-2.77) P=0.245 | OR=0.98 (0.62-1.56) P=0.941 | OR=1.78 (0.99-3.19) P=0.055 | OR=0.68 (0.35-1.23)  P=0.199 | OR=0.55 (0.34-0.89) P=0.017 | OR=0.56 (0.28-0.69)  P<0.001 | OR=0.78 (0.41-1.47) P=0.442 | OR=1.78 (0.44-2.00) P=0.872 | OR=0.56 (0.43-3.13) P=0.771 |

*composite of death, stroke, myocardial infarction, major vascular complication, major bleeding, or renal failure; OR=odds ratio (<1 favors first treatment, >1 favors second treatment); β=regression coefficient (<0 favors first treatment, >0 favors second treatment); all other endpoints were not significantly different or inconclusive given sparse data

**Table 4S.** Breakdown of procedures according to center overall volume, included cases, and device type.

| **Center** | **Total procedures*** | **Procedures selected*** | **Acurate†** | **Evolut†** | **Lotus†** | **Portico†** | **Sapien3†** |
| --- | --- | --- | --- | --- | --- | --- | --- |
| 3 | 438 (12.5%) | 384 (19.4%) | 27 (7.0%) | 181 (47.1%) | 52 (13.5%) | 1 (0.3%) | 123 (32.0%) |
| 4 | 90 (2.6%) | 48 (2.4%) | 0 | 17 (35.4%) | 18 (37.5%) | 13 (27.1%) | 0 |
| 10 | 109 (3.1%) | 14 (0.7%) | 0 | 9 (64.3%) | 1 (7.1%) | 0 | 4 (28.6%) |
| 11 | 459 (13.1%) | 291 (14.7%) | 12 (4.1%) | 119 (40.9%) | 0 | 160 (55.0%) | 0 |
| 12 | 2 (0.1%) | 1 (0.1%) | 0 | 0 | 0 | 0 | 1 (100%) |
| 14 | 41 (1.2%) | 14 (0.7%) | 0 | 13 (92.9%) | 0 | 0 | 1 (7.1%) |
| 15 | 381 (10.9%) | 280 (14.2%) | 0 | 98 (35.0%) | 0 | 0 | 182 (65.0%) |
| 17 | 229 (6.5%) | 186 (14.2%) | 65 (35.0%) | 91 (48.9%) | 4 (2.2%) | 0 | 26 (14.0%) |
| 20 | 16 (0.5%) | 15 (0.8%) | 1 (6.7%) | 4 (26.7%) | 0 | 0 | 10 (66.7%) |
| 21 | 399 (11.4%) | 227 (11.5%) | 49 (21.6%) | 26 (11.5%) | 13 (5.7%) | 4 (1.8%) | 135 (59.5%) |
| 22 | 4 (0.1%) | 1 (0.1%) | 1 (100%) | 0 | 0 | 0 | 0 |
| 24 | 82 (2.3%) | 38 (1.9%) | 0 | 1 (2.6%) | 0 | 0 | 37 (97.4%) |
| 27 | 20 (0.6%) | 9 (0.5%) | 0 | 0 | 9 (100%) | 0 | 0 |
| 28 | 363 (10.4%) | 299 (15.1%) | 79 (26.4%) | 10 (3.3%) | 54 (18.1%) | 134 (44.8%) | 22 (7.4%) |
| 29 | 49 (1.4%) | 42 (2.2%) | 0 | 8 (18.6%) | 0 | 35 (81.4%) | 0 |
| 30 | 129 (3.7%) | 126 (6.4%) | 0 | 126 (100%) | 0 | 0 | 0 |

*per-row percentages are reported; †per-column percentages are reported

**Table 5S.** Selected one-month outcomes according to overall and device-specific volume.

| **Feature** | **Overall TAVI volume** | **Acurate volume** | **Evolut volume** | **Lotus volume** | **Portico volume** | **Sapien3 volume** |
| --- | --- | --- | --- | --- | --- | --- |
| Major adverse event* | P=0.018^#^ | P=0.097 | P=0.435 | P=0.205 | P=0.101 | P=0.217 |
| Death | P=0.020^#^ | P=0.341 | P=0.675 | P=0.010^#^ | P=0.459 | P=0.847 |
| Myocardial infarction | P=0.022^#^ | P=1 | P=0.851 | P=1 | P=1 | P=0.406 |
| Stroke or transient ischemic attack | P=0.739 | P=0.834 | P=0.887 | P=0.339 | P=0.460 | P=0.889 |
| Major vascular complication | P=0.133 | P=0.271 | P=0.830 | P=0.500 | P=0.226 | P=0.601 |
| Major bleeeding | P=0.058 | P=0.531 | P=0.293 | P=0.084 | P=0.435 | P=0.491 |
| Renal failure | P=0.163 | P=0.108 | P=0.074 | P=0.415 | P=0.203 | P=0.163 |
| Permanent pacemaker | P=0.009† | P=0.272 | P=0.371 | P=0.068 | P=0.542 | P=0.239 |

*composite of death, stroke, myocardial infarction, major vascular complication, major bleeding, or renal failure; †favoring low volume centers (i.e. centers in the lowest tertile of volume); ^#^favoring high volume centers (i.e. centers in the highest tertile of volume) **Table 6S.** One-month major adverse events* propensity score-adjusted analysis adjusting for overall and device-specific volume.

| **Adjustment** | **Acurate vs Evolut** | **Acurate vs Lotus** | **Acurate vs Portico** | **Acurate vs Sapien3** | **Evolut vs Lotus** | **Evolut vs Portico** | **Evolut vs Sapien3** | **Lotus vs Portico** | **Lotus vs Sapien3** | **Portico vs Sapien3** |
| --- | --- | --- | --- | --- | --- | --- | --- | --- | --- | --- |
| Overall volume | P=0.903 | P=0.333 | P=0.012# | P=0.765 | P=0.757 | P=0.155 | P=0.045† | P=0.318 | P=0.134 | P=0.125 |
| Volume with Acurate | P=0.741 | P=0.297 | P=0.211 | P=0.811 | - | - | - | - | - | - |
| Volume with Evolut | P=0.840 | - | - | - | P=0.963 | P=0.015# | P=0.025† | - | - | - |
| Volume with Lotus | - | P=0.246 | - | - | P=0.413 | - | - | P=0.660 | P=0.115 | - |
| Volume with Portico | - | - | P=0.057 | - | - | P=0.544 | - | P=0.985 | - | P=0.278 |
| Volume with Sapien3 | - | - | - | P=0.587 | - | - | P=0.538 | - | P=0.135 | P=0.590 |

*composite of death, stroke, myocardial infarction, major vascular complication, major bleeding, or renal failure; †favoring the first device; ^#^favoring the latter device

**Figure 1S.** Key baseline and procedural differences in patients undergoing transcatheter aortic valve implantation with Acurate, Evolut, Lotus, Portico and Sapien3, with ↑ indicating higher values/rates, = indicating similar ones, and ↓ indicating lower ones.


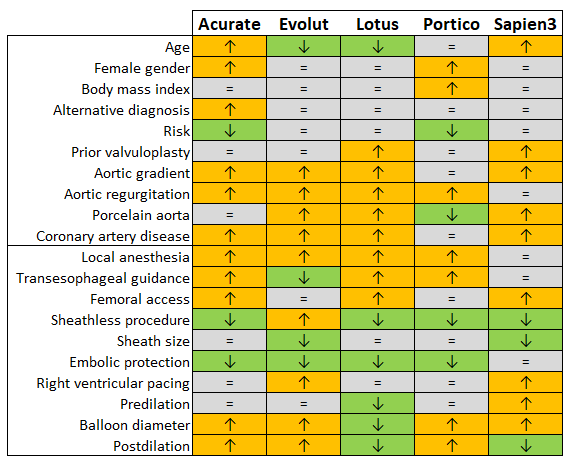

Supplement: Supplementary file 1 — Supplementary Information [file 41598_2019_53081_MOESM1_ESM.docx]
